# Disseminated mucormycosis (DM) after pneumonectomy: a case report

**DOI:** 10.1186/s12879-016-1639-3

**Published:** 2016-07-22

**Authors:** Qian Wang, Bo Liu, Youde Yan

**Affiliations:** Department of Infectious Diseases, The First Affiliated Hospital with Nanjing Medical University, 300 Guangzhou Road, Nanjing, 210029 Jiangsu China; Department of Infection Management, The First Affiliated Hospital with Nanjing Medical University, Nanjing, 210029 China

**Keywords:** Case report, Disseminated mucormycosis, Pneumonectomy, Immunocompetent

## Abstract

**Background:**

Mucormycosis is a kind of rare opportunistic fungal disease and the incidence of which has gradually increased. Disseminated mucormycosis (DM) is a life-threatening infection that mostly occurs in immunocompromised patients. The lung and brain are usually involved in disseminated mucormycosis, and other sites are scare. We report the first case of disseminated mucormycosis whose infection sites included lung, skin, liver, vertebra, and spinal cord that ensued after a right lung pneumonectomy in an immunocompetent patient.

**Case presentation:**

A 20-year-old female underwent a right lung pneumonectomy for “lung cancer” presented with an intermittent fever for two years. A computed tomography (CT) scan showed an enclosed outstanding mass in the right chest wall. The patient also suffered from lower limb numbness and weakness, difficulty walking, and dysuria. Medical examination showed superficial feeling of the abdominal wall was decreased from the T7 and T8 level; muscle strength for both lower limbs was decreased; muscle tension of both lower limbs was also diminished. A biopsy through the right chest wall mass and thoracic mass by fistula of chest wall showed broad nonseptate hyphae with right-angle branching, consistent with mucormycosis. With titration of amphotericin B and its lipid complex, the patient recovered.

**Conclusions:**

Our case showed an unusual clinical presentation of disseminated mucormycosisin an immunocompetent patient.

## Backgroud

Mucormycosis is caused by fungi of the order Mucorales. The most common kinds of the order Mucorales which cause human infections include *Rhizopus spp., Mucor spp., Lichtheimia spp., Rhizomucor spp.* and *Apophysomyces spp.*, especially the third members accounting for 70–80 % of all infectious cases [1]. Mucormycosis is a life-threatening infection that carries a high mortality rate, despite recent advances in its diagnosis and treatment [2]. It often occurs among immunocompromised patients, such as those with hematological malignancies, solid organ transplantation, or diabetes mellitus. Based on anatomic localization, mucormycosis can be classified as rhinocerebral, pulmonary, cutaneous, gastrointestinal, disseminated, or uncommon presentations [3].

Disseminated mucormycosis (DM) often involves two or more noncontinuous organ systems with zygomycetes in immunocompromised patients with hematological malignancies or organ transplantation, or in patients on deferoxamine therapy [4, 5]. The lung and brain are usually involved in DM, however, other sites are scare. In this report, we describe a case of DM in an immunocompetent young woman who had a right lung pneumonectomy almost two year prior.

## Case presentation

**Fig. 1:**
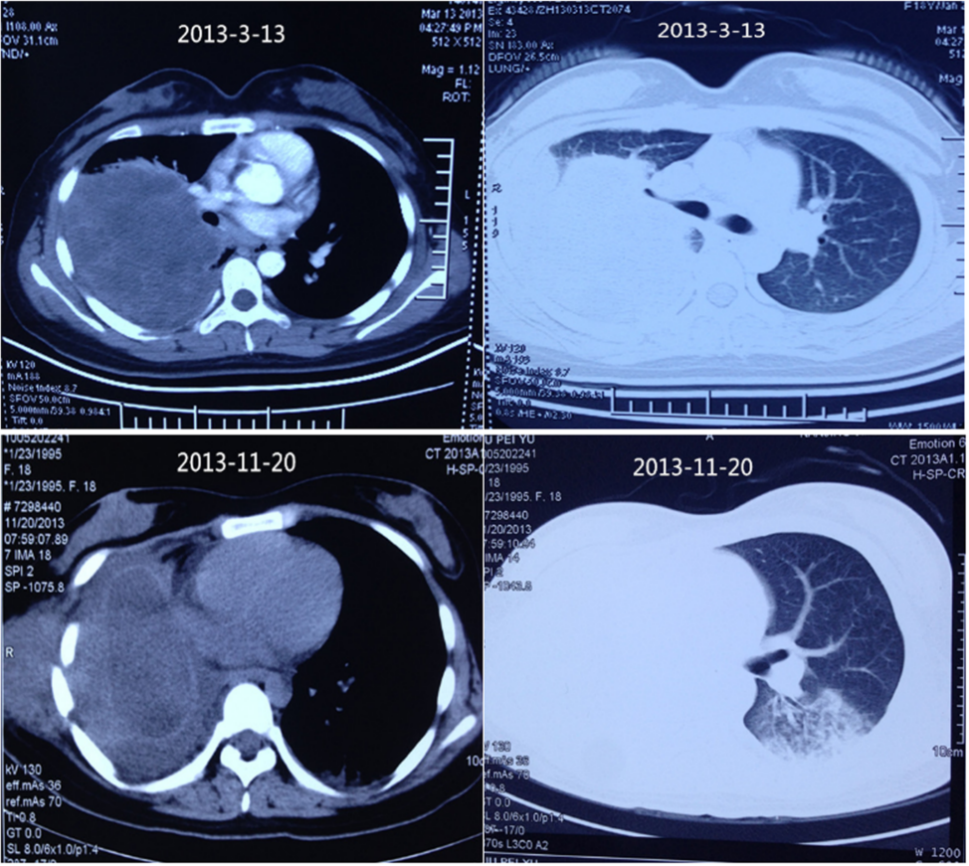
The computed tomography (CT) scan showed an enclosed outstanding mass in the right chest wall 7-month later after the right lung resection

A 20-year-old female who presented with an intermittent fever for two years and double lower limb numbness and weakness for one month was admitted to hospital in January 2015. In April 2013, she underwent a right lung pneumonectomy for “lung cancer,” while the postoperative pathology referred to a “necrotizing granulomatous mass.” Seven months after the operation, a computed tomography (CT) scan showed an enclosed outstanding mass in the right chest wall (Fig. 1). Thereafter, the patient was hospitalized three times due to a repeated fever and multiple bump biopsies from the enclosed outstanding mass; however, there was no clear diagnosis. One month before this admission, in addition to a fever, the patient suffered from worsened lower limb numbness and weakness, difficulty walking, and dysuria. In the past, she was healthy and had good nutrition.

**Fig. 2:**
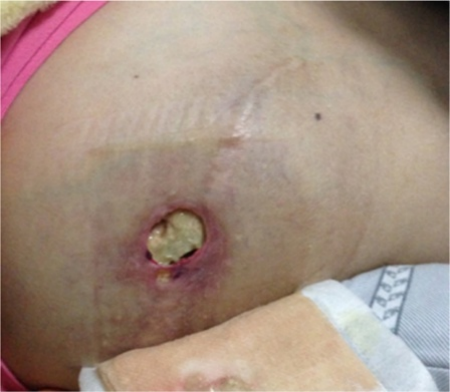
Thoracic swelling with a protruding mass from the right chest wall was observed. The central part of the pack was a 0.3-cm-diameter fistula, which oozed a nonodorous, yellowish turbid liquid

A physical examination showed an abnormal and oncotic hard mass (10 cm × 5 cm) that protruded from the right chest wall. A 0.3-cm-diameter external fistula was seen in the central part of the pack (Fig. 2), which oozed a nonodorous, yellowish turbid liquid. Coarse sounds without moist rales or rhonchi were heard with left lung auscultation. Superficial feeling of the abdominal wall was decreased from the T7 and T8 level, while a deep feeling was present. Muscle strength for both lower limbs was decreased: 1° right and 3° left. Muscle tension of both lower limbs was also diminished. The bilateral Babinski sign was positive, but concavity swelling of the lower limbs existed.

Laboratory tests showed a white blood cell count of 18.21 × 10^9^/L, a hemoglobin level of 82 g/L, a platelet level of 509 × 10^9^/L, a C-reactive protein concentration of 98.00 mg/L, an erythrocyte sedimentation rate of 98.00 mg/L, and negative 1-3-β-D glucan and glactomannan (GM) tests. Post right pneumonectomy, the thoracic CT scan revealed uneven density flake soft tissue shadows in the right chest cavity, multiple sizes of nodules in the left lung, a soft tissue mass (12.5 cm × 5.9 cm) around a small pneumatosis in the right chest wall, and a reduced density in the right liver lobe near the diaphragmatic top (January 2015, Fig. 3). The thoracic magnetic resonance imaging (MRI) results showed that a space-occupying lesion existed in the right pleural and chest wall with the T3-6 vertebral body and the right rib damaged, invading the spinal channel and spinal cord (January 2015, Fig. 3).

**Fig. 3:**
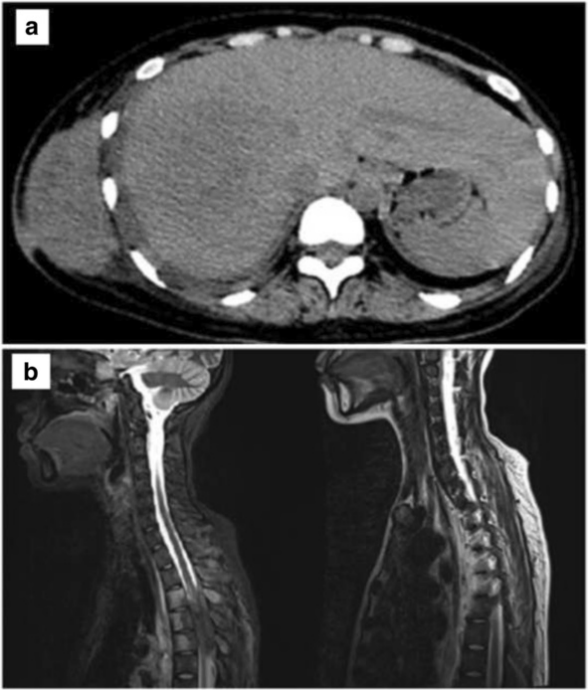
Results of CT and MRI (January 2015) for the chest. The CT shows a soft tissue mass (12.5 cm × 5.9 cm) around a small pneumatosis in the right chest wall, and a reduced density in the right liver lobe near the diaphragmatic top (**a**). The MRI shows lesions invaded the spinal channel and spinal cord (**b**)

**Fig. 4:**
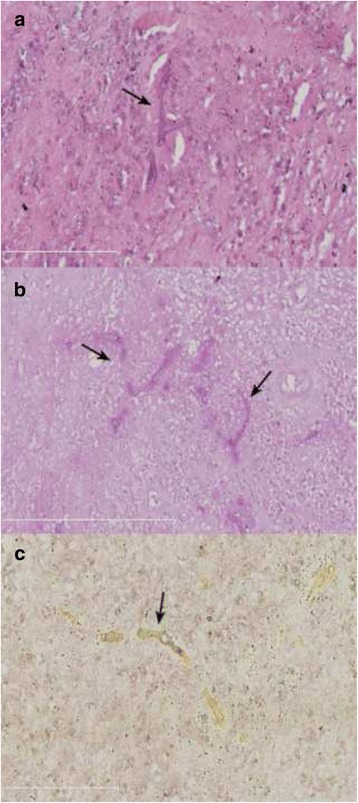
Histopathology showed broad nonseptate hyphae with right-angle branching, **a** (HE × 400), **b** (PAS × 400) and (**c**) (silver impregnation × 400)

A biopsy through the right chest wall mass and thoracic mass by fistula of chest wall showed broad nonseptate hyphae with right-angle branching, consistent with mucormycosis (Fig. 4). The mucormycosis was widely disseminated, invading the lung, skin, liver, vertebrae, and spinal cord.

The patient quickly developed type II respiratory failure. A noninvasive ventilator assisted breathing, and intravenous amphotericin B (AmB) was administered. When the dose of AmB increased to 0.5 mg/kg/day, with the cumulative dose reaching 150 mg, mental disorders (agitation, fear, etc.) and cardiac arrhythmias occurred. The adverse events disappeared when AmB was changed to liposomal AmB.

After treatment for 28 days, the patient’s body temperature became normal and her respiratory function as well as lower limb sensory and motor function recovered. The size of the right chest wall mass became smaller, and the fistula dried. Laboratory and imaging examinations indicated that the lesions of the lung, liver, and thoracic vertebrae were significantly smaller. The patient was discharged back to the local hospital for further antifungal treatment and had recovered by the 3-month follow-up exam, and plastic surgery was suggested to repair the thoracic wall.

## Discussion

In this report, we present a case of DM in the lung, skin, liver, vertebrae, and spinal cord after a right lung pneumonectomy. The lung and brain are usually involved in DM [6]. The involvement of the vertebrae and spinal cord in our case has rarely been reported in DM. DM has a nonspecific manifestation, resulting in difficulties in diagnosis [5]. In our case, it was not difficult to speculate that it first occurred in the lung, even before the pneumonectomy, as a necrotizing granulomatous mass was found. Indeed, pulmonary infection is the second most common form, accounting for more than 30 % of infections [7]. Mucorales has a special affinity to endothelial cells, resulting in its invasion and destruction of small arteries. By attracting platelet adhesion and aggregation, Mucorales can also cause thrombosis and fatal bleeding [8, 9]. This might explain the wide dissemination of pulmonary mucormycosis in the present case.

To date, there are no known reliable clinical serological diagnostic methods for the diagnosis of DM. Microbial culture of secretions is time-consuming, with a low sensitivity and a high false-positive rate. The culture of sterile tissue may kill the pathogen because of its grinding process (a step for preparing tissue culture samples), which results in a low rate of positive cultures [10]. Therefore, the gold standard for the diagnosis of DM relies on the characteristic mycelium and pathological changes of the biopsy. The histopathological characteristics for Mucorales is a wide (6–25) μm in diameter, rarely separating hyphae and have irregular or right angle branch. The diagnosis of pulmonary mucormycosis in this report was based on histopathology.

DM generally occurs in severely immunocompromised patients: it represents 1.6 % of all invasive fungal infections and is predominant in immunosuppressed patients with risk factors [11]. Recently, the incidence has increased significantly, even in immunocompetent patients [11]. Our report describes DM in an immunocompetent patient. DM is associated with a mortality rate of approximately 100 %, but successful treatment has been reported [12–14]. Successful treatment of DM requires a rapid diagnosis, reversal of predisposing factors, aggressive surgical excision, and antifungal therapy [15]. AmB or its lipid complex remains the first choice for treatment [16]. In the present case, after titrating the dose to avoid adverse effects, the patient recovered quickly.

## Conclusion

Clinicians need raise awareness that DM should also be regarded in immunocompetent individuals, especially those with pulmonary mucormycosis. Retrospective studies regarding the morbidity of DM in immunocompetent individuals in the future might be necessary in order to understand its epidemiology.

## Abbreviations

AmB, amphotericin B; CT, computed tomography; DM, disseminated mucormycosis; GM, glactomannan; MRI, magnetic resonance imaging
